# Does the Formulation of the Decision Problem Affect Retirement?—Framing Effect and Planned Retirement Age

**DOI:** 10.3390/ijerph19041977

**Published:** 2022-02-10

**Authors:** Tomasz Jedynak

**Affiliations:** Department of Risk Management and Insurance, Cracow University of Economics, 31-510 Kraków, Poland; jedynakt@uek.krakow.pl

**Keywords:** framing effect, pensions, retirement age, determinants of retiring, retirement decisions, behavioral aspects of retiring, behavioral economics, psychology of decision-making

## Abstract

The aim of the study is to fill the research gap in relation to one of the behavioral factors that have a potential impact on retirement decisions—the framing effect. A research question addressed in the study is whether the way in which the decision-making problem is formulated (the framing effect) influences decisions on the planned retirement age. To answer this question, an original research questionnaire was developed. It included a description of a hypothetical pension system and experimental vignette questions. The research was conducted on the basis of answers given by 1079 randomly selected respondents who were participants of the pension system in Poland before retirement. In the analysis of the results, non-parametric tests and multiple logistic regression were used to compare response distributions. As a result of the conducted research, it was proven that the framing effect significantly affects the extension of the planned retirement age. At the same time, it was found that loss framing affects pension decisions to a greater extent than gain framing. It has also been noted that women are more susceptible than men to the framing of pension decisions. An application conclusion resulting from the conducted research is indicated as the possibility of the intentional use of the framing effect by decision-makers in order to increase the effective retirement age.

## 1. Introduction

Demographic change is one of the biggest societal challenges facing developed countries. The functioning of old-age security systems is an area in which the consequences of the ongoing changes are particularly visible. Therefore, in recent years, both in the public debate and scientific discourse, the issue of pension systems has been raised increasingly often. Alongside the financial stability of pension systems and income adequacy of pensions, the issue of retiring and retirement age are one of the most important topics of current debates.

The growing interest in the issue of retirement age results from several issues. Firstly, in an ageing society and with shrinking labor force resources, it is highly desirable to extend the working lives of older people. Secondly, delaying retirement has a positive impact on the adequacy of pension benefits and, taken as an aggregate, improves the financial sustainability of pension systems. Thirdly, many developed countries have already raised or intend to raise the statutory retirement age: according to the OECD, 21 out of 36 member states have raised or intend to raise the statutory retirement age. At the same time, in many countries there is a widening gap between the statutory and effective retirement age [1].

In economic literature, the issues of retirement age and retiring are usually embedded in the neoclassical approach to economics and are analyzed from a macro perspective. In this study, these issues are analyzed through the prism of the achievements of behavioral economics, from a micro perspective.

The adoption of a microeconomic perspective in the analysis of pension issues is based on the concept of a life cycle model [2,3,4], which implies treating the pension system as a tool for allocating income (smoothing consumption) during the life of an individual [5,6]. This takes into account three basic elements that determine an individual’s participation in the pension scheme: (1) the accumulation of savings (pension rights) during the period of professional activity; (2) the payment of these savings during retirement; and (3) the moment separating these two periods, i.e., the age of concluding professional activity and beginning retirement. The indicated elements cover the most important parameters of the pension system and as such are key areas of pension decisions. These decisions can be taken both by the state and individually by each participant in the pension system; however, the decision-making nature of individuals is limited by applicable law. Among the listed areas of pension decisions, the focus of the considerations presented in this study are individual decisions regarding the choice of the moment of retirement from work.

At the root of the research problem is the question of what influences individual decisions regarding the choice of a specific moment of retirement. In other words, why do different people decide to end employment and retire at different ages? Traditionally, the answer to this question is sought in neoclassical pension models and empirical research based on them (see the review of research in Section 2.1). Meanwhile, in the field of pension economics, as in the whole of economics, the behavioral approach initiated by D. Kahneman and A. Tversky [7,8,9], further developed by authors such as R. Thaler [10,11] is becoming increasingly important. This approach involves incorporating psychological tools and insights into the study of economic problems [12]. Behavioral economics understood in such a manner, according to C. Camerer and G. Loewenstein, enhance the explanatory power of economics by providing them with more realistic psychological foundations [13].

In the area of pension economics, the behavioral approach has been present since the beginning of the 21st century. However, research in this area has focused mainly on the issue of accumulating retirement savings and, less frequently, on the issue of disbursement of these savings [14]. Relatively less space is devoted to the behavioral conditioning of retirement decisions. Hence, the main point of this study is to fill this research gap. The article focuses on one of the identified potential behavioral factors that have a potential impact on retirement decisions: the impact of the way the decision-making problem is presented (the framing effect).

The framing effect is a well-analyzed and empirically proven cognitive bias. It focuses on how the presentation of the decision-making situation impacts the choices of decision-makers. As a rule, people tend to avoid risk when a positive frame is presented (i.e., a frame emphasizing benefits) and tend to take risks when a negative frame (i.e., a frame accentuating losses) is presented [9,15]. With regard to the decision to retire, the framing effect can be defined as the impact of presentations of the decision-making situation (choice of retirement age and the related amount of retirement benefits) on decisions on the retirement age (actual or planned).

Suspecting that, as in other areas of decision-making, and in the field of retirement, the framing effect may play an important role, a research problem addressed in this study was formulated. It is expressed by the question: does the way in which the decision-making problem is formulated (the framing effect) influence decisions on the planned retirement age? Based on the indicated research problem, detailed objectives of the study are formulated. The theoretical objectives are: to identify and discuss the behavioral determinants of the decision to retire and to characterize the framing effect in the context of these decisions. The methodological objective is to develop a research methodology to quantify the impact of the framing effect on retirement decisions. The primary research objective is to assess the impact of the framing effect on decisions regarding the planned retirement age.

Presenting the contribution of this article to the literature, first of all it should be pointed out that, unlike most previous studies dealing with the issue of behavioral determinants of retirement decisions, it does not focus on the issue of saving for retirement but takes up the topic of choosing the moment of retirement. Within this thread, the contribution of the presented study has a threefold character: theoretical, methodical and empirical. The theoretical gap to be filled consists of presenting the mechanism of operation of the framing effect as a behavioral factor that affects individual decisions about retirement. The methodological contribution centers on the development of an original method of measuring and assessing the impact of the framing effect on retirement decisions. In particular, a unique solution is the use in this area of the description of a hypothetical pension system and vignette questions based on it. In the empirical aspect, the study presents the results of using the original research procedure to assess the impact of the framing effect on the planned retirement age of non-retired participants of the Polish general pension system.

## 2. Literature Review—Neoclassical and Behavioral Determinants of Retiring

### 2.1. The Neoclassical Approach to Retirement Decisions

The economic literature regarding the determinants of the decision to retire is rich and on the surface seems to provide a comprehensive answer to the question posed in the introduction, regarding the factors influencing individual decisions about retirement. As part of the theoretical trend of research, on the basis of the theory of saving and consumption, since the 1980s various types of pension models have been built, which are intended to describe the decision-making process leading to the decision to end professional activity [16,17,18]. Among the most well-known pension models are: simple one-period models of choice between work and leisure [17,19,20]; static life cycle models [16,21,22,23,24]; universal Fields-Mitchell model [25,26]; more complex dynamic life cycle models [27,28] and option pricing models [29,30,31,32]; as well as the most mathematically advanced stochastic dynamic programing models [33,34,35,36,37,38,39]. These elements, in addition to presenting rational reasoning leading to the decision to retire, also make it possible to identify the factors that are taken into account by individuals when making decisions about starting their retirement.

The identification and determination of the direction and strength of the influence of these factors on pension decisions have been the subject of many empirical studies over the past half century representing the neoclassical trend of research on retirement decisions. A review of the literature leads to the distinction of a number of conditions that may influence individual decisions regarding retirement. For example, M. Scharn et al. [40] listed 49 factors potentially influencing the moment of retirement. R. Lumsdaine and O. Mitchell [18], reviewing the various factors influencing retirement decisions, distinguished: economic factors, health problems and disabilities, institutional limitations, family considerations and caring responsibilities. An extensive meta-analysis of the various factors influencing retirement decisions was also carried out by G. Fisher et al. [41].

Research to date shows that the factors that have a significant impact on retirement decisions are, in particular, financial factors such as the level of remuneration [24,42], the amount of retirement benefits, the generosity of the pension system [19,43,44,45,46] and the amount of pension wealth [47,48,49,50]. Pension decisions are also influenced by factors related to the design of the social security system [51,52,53]; including the statutory retirement age [36,44,54,55,56,57] and the ongoing reforms of pension systems [58]. Factors of an individual nature include: health status [22,39,50,59,60,61,62,63,64]; life expectancy [65,66]; characteristics of work and occupation [59,67,68,69]; job satisfaction [63,70,71,72]; marital status and family conditions, including caring responsibilities [50,73,74,75]. The decision to retire may also be influenced by the wish for more leisure [64]. In addition, research shows that decisions on retirement are influenced by macroeconomic variables, such as unemployment levels [76,77,78] and the overall economic and market situation [79,80,81,82]. Among the various determinants affecting the effective retirement age, the demographic situation is also indicated (e.g., [83]). Another important issue raised in research to date is the impact of retirement on overall well-being and quality of life [84].

### 2.2. Behavioral Determinants of Retirement Decisions

The above-mentioned research on the factors influencing the decision to retire was conducted within the framework of the mainstream, neoclassical economics. They therefore focused on factors formulated on the basis of models of retirement, which are based on the paradigm of the rational man (homo economicus). However, the discoveries within behavioral economics suggest that, in the context of making individual decisions about retirement, as is the case in other fields of interest in economics and finance, the legitimacy of this paradigm should be questioned [13]. A number of studies prove that people in their choices do not behave as rationally as economists would like (e.g., [9,11]). It is also no different in the case of decisions regarding participation in the pension system. 

The observation of actual retirement decisions leads to the identification of a whole catalogue of behaviors that cannot be fully explained by neoclassical pension models. For example, it is indicated that individuals do not retire at the moment defined in these models as optimal and do not take into account the maximization of their expected life utility. This phenomenon, visible at the individual level, due to the mutual elimination of deviations, often escapes analysis at the aggregate level [85]. Among the observable deviations from optimal—according to neoclassical models—retirement behaviors, researchers mention:

- Basing decisions on retirement on the default options: the minimum retirement age or the universal retirement age [86,87],
- Non-accession of eligible persons to pension schemes offering economically advantageous conditions of participation linked to employer-funded subsidies [88],
- Choosing sub-optimal portfolios of retirement investments: investing retirement savings in products that do not minimize fees [89,90],
- Making pension decisions despite a lack of knowledge of pension rules [91],
- Basing pension decisions on the behavior of people from the social environment and social norms [92,93].

In addition, the differences in retirement savings between households with similar socio-economic characteristics depend only slightly on differences in: rates of time preference, tendency towards risk, preference for work and leisure and replacement rates [94]. An interesting fact that supports the thesis about the lack of rationality of pension decisions is also the fact that Americans retire more often than the statistics themselves would indicate on their birthday [95].

Therefore, looking for a comprehensive answer to the question posed earlier, about what influences individual decisions regarding retirement, in addition to factors occurring in neoclassical pension models, one should also refer to behavioral conditions. It is indicated that the observed deviations from model pension decisions can be explained by the concepts of behavioral economics, such as: heuristics, perspective theory, behavioral life cycle hypothesis, mental accounting or hyperbolic discounting [92,96]. At the same time, it should be emphasized that the behavioral approach does not deny the impact of neoclassical factors on pension decisions. On the contrary, these factors are taken as a starting point for in-depth analyses of retirement decisions, which additionally take into account behavioral determinants. The behavioral approach is intended to expand, not replace, mainstream economics.

As already mentioned in the introduction, in retirement economics to date, the behavioral approach has focused primarily on the area of accumulation of retirement savings (e.g., [88,97,98,99,100,101,102,103,104]) and, to a lesser extent, the payment of these savings [105,106]. Numerous studies conducted in this area show that savings decisions made by future retirees are significantly influenced by behavioral conditions, such as: changing the choice architecture by introducing an automatic enrollment mechanism [88,107,108], automatic escalation of the contribution [100] as well as simplifying the investment selection process by reducing the number of available options and introducing default options [14,88,102,103].

The behavioral context of the decision to retire has so far been given little space in the literature. The few theoretical and conceptual studies dealing with this theme include the works of M. Knoll [96] and F. Erp, N. Vermeer and D. van Vuuren [92]. On the empirical side, this issue was considered by D. Fetherstonhaugh and L. Ross [109]; J. Liebman and E. Lutmer [110,111]; L. Behaghel and D. Blau [112]; J. Brown, A. Kapteyn and O.S. Mitchell [113]; and N. Vermeer, M. van Rooij and D. van Vuuren [93]. The implications of these studies suggest that decisions about retirement may be influenced by behavioral factors such as: (1) the framing effect; (2) default options; (3) the anchoring effect; (4) impact of social norms and the social environment; (5) hyperbolic discounting; (6) planning fallacy and (7) affective forecasting.

The synthetic characteristics of these behavioral factors are described in Table 1. The framing effect, which is the main subject of research undertaken in this work, is referred to in more detail in the next section.

### 2.3. Framing Effect and the Decision to Retire

According to the assumption of standard economic models, the way the decision-making problem is formulated should not affect the decisions made. A rational decision-maker always strives to maximize his/her satisfaction (utility) guided by a specific system of preferences. In reality, however, this assumption is often wrong. As evidenced by the results of research by behavioral economists, the way the decision-making problem is presented can have a key impact on the choices made in different areas of life [15,115,116,117,118,119,120].

In pension economics, one of the most prominent examples of the impact of the formulation of a decision-making problem on the decision-making, concerns the problem of accumulating savings for retirement. B. Madrian and D. Shea [88] surveyed participants in the American 401(k) retirement program and showed that the level of participation in supplementary pension programs largely depends on whether the decision to participate in these programs is presented in the form of a positive selection or in the form of automatic enrolment. In the first case, the level of participation was 37%, in the second, 86%. S. Benartzi and R. Thaler [97,99] in turn proved the ways in which the different options for selection regarding the composition of the portfolio of investments in the pension scheme have a greater impact on the choice of the participant than the rates of return and risk associated with the individual investment options. In other studies, the same authors [121] have shown that investment decisions are influenced by the way information is presented. Depending on whether the pension plan members were presented with annual rates of return on shares or more stable 30-year rates, the average share of shares in their portfolios differed by almost 18 p.p.

With regard to decisions on retirement, the framing effect boils down to the impact of the way in which the decision-making situation is presented on the choices of decision-makers regarding their actual or planned retirement age [85]. It is worth illustrating the operation of this effect with an example. Let us consider the following three decision-making problems:

- $P_{0}$: retirement at the age of $R_{65}$ (65 years) entails a retirement pension of $B_{65}$. Earlier or later retirement will result in a decrease or increase in this benefit by amount X for each year of difference. At what age to retire?
- $P_{1}$: retirement at the age $R_{66}$ (66 years) entails a pension greater by *G*, than in the case of retiring at age $R_{65}$. At what age to retire?
- $P_{2}$: retirement at age $R_{65}$ (65 years) entails a pension reduced by *L,* less than in the case of retiring at age $R_{66}$. At what age to retire?

Assuming that X = G = L, the problems $P_{0},\;P_{1}$, $P_{2}$ are identical in content and differ only in the form of the description of the situation. By acting rationally, the decision-maker should therefore make the same decision in every case.

However, taking into account the conclusions of the previous achievements of behavioral economics, in particular those regarding the operation of the framing effect, it can be assumed that depending on the way the decision-making problem is formulated, people will make different choices. In particular, bearing in mind loss aversion, it can be assumed that if retirement at the age of $W_{1}$ is presented in the category of losses in relation to a specific reference age $W_{2}$ (problem $P_{2}$), then more people will decide to extend their professional activity than in the base variant $P_{0}$ that does not take framing into account.

The assumptions formulated above are reflected in the research hypotheses formulated in the next section. Importantly, the validity of these hypotheses is partly confirmed by the results of previous empirical studies. The existence of a framing effect in the area of pension decisions has been confirmed by D. Fetherstonhaugh and L. Ross [109], who presented respondents with differently formulated hypothetical problems regarding the choice of the moment of retirement. They found that, when the opportunity to claim retirement benefits at age 68 was presented as resulting in profit relative to the benchmark (65), only 38% of those surveyed chose 68 as their preferred retirement age. On the other hand, when receiving benefits at the age of 65 was determined to result in a loss compared to the reference point at the age of 68, as many as 57% of respondents chose 68 as the preferred retirement age. At the same time, however, D. Fetherstonhaugh and L. Ross did not note a similar effect in the case of differently presented alternatives to retirement at the age of 62 or 65.

A similar experiment was conducted by J. Liebman and E. Luttmer [110]. They presented to three groups of respondents the situation regarding retirement at the age of 62 or 65, formulated in three different ways (loss framing, gain framing and break-even frame). It turned out that the delay in applying for a retirement benefit was declared by: 56% of people who were presented with a framework emphasizing losses, 64% of people who were presented with a framework emphasizing profits and 46% of people who were presented with a framework in the form of a break-even point.

Evidence that framing influences retirement decisions is also provided by research by J. Brown, A. Kapteyn and O.S. Mitchell [113]. The results of their experiment suggest that framing is important when making decisions about retirement. The authors note that: (1) the break-even frame results in an earlier expected age of claiming a retirement benefit (approx. 19–22 months) compared to the baseline scenario; (2) framing that accentuates losses leads to an earlier expected claim for a benefit (approx. 2–5 months); (3) an age anchor at the level of 62 years reduces the expected age of applying for the benefit (by approx. 2–6 months), and an anchor at the level of 70 years results in an increase in this value (by approx. 1–4 months).

On the slightly different side, the framing effect was studied by C. Merkle, P. Schreiber and M. Weber [122]. These authors linked the decision on the time of retirement to the discrepancy between the presentation in the form of willingness-to-accept (the minimum increase in monthly benefits inducing people to delay retirement) and in the form of willingness-to-pay (the maximum and the amount of monthly benefits, which people are willing to give up to take early retirement). According to the results obtained by the researchers, the way in which the decision-making problem was formulated in one of these frameworks significantly influenced the decisions of future pensioners. In particular, compared to the willingness-to-pay framework, the willingness-to-accept framework influenced the tendency of Germans to retire early (in this case, the implicit probability of earlier retirement increased by 30 p.p.).

## 3. Materials and Methods

### 3.1. Research Hypothesis

The main research problem raised in this paper is outlined in the introduction. It was expressed in the form of a question about whether the way the decision-making problem is formulated affects decisions regarding the planned retirement age. On the basis of the literature review and in-depth theoretical studies, three research hypotheses were put forward in relation to the problem formulated in this way:

**Hypothesis** **1** **(H1).** *The framing of the decision-making problem of retirement influences the extension of the planned retirement age.*

**Hypothesis** **2** **(H2).** *Loss framing extends the planned retirement age more than gain framing.*

**Hypothesis** **3** **(H3).** *The broader framework of the decision-making problem affects the extension of the planned retirement age more than the narrow framework.*

Verification of such research hypotheses was carried out on the basis of a survey conducted among non-retired participants of the Polish pension system. The implementation of this study required the development of an original research methodology, which included the operationalization of the concepts of planned retirement age and retirement, the development of a research questionnaire and the definition of research scope.

### 3.2. Planned Retirement Age and Decision to Retire

In this study, the impact of the framing effect on retirement decisions was not considered directly on the basis of an analysis of actual retirement decisions, but through the prism of the planned retirement age. Analyses of the planned retirement age are common in research practice [46,59,73,123], which is also used in some studies on the behavioral determinants of retirement decisions [87,93,113].

The justification for the approach based on planned retirement age is the imperfection of analyses based on real choices. Analyses of this kind can be used only to examine the effects of minor deviations from the actual situation. On the other hand, studies based on declared preferences can also be used in research relating to choice in conditions that differ significantly from the actual situation [46]. The study of the planned retirement age also enables an isolated analysis of the impact of behavioral factors on these decisions and allows the use of elements of a controlled experiment in the research. The analysis of planned retirement age also has a practical justification: it is much faster and cheaper than the study of real retirement decisions, which requires a specific selection of the research sample and to be spread over time.

Considering retirement, E. Lazear [17], among the possible objective definitions of this concept, points to situations in which a person: (1) is outside the labor force with the intention of remaining outside it permanently; (2) has significantly reduced his/her working hours compared to a certain lifetime average and intends to keep the working hours at or below this level; (3) receives part of the income as a retirement pension; (4) appears on the list of pensioners in the occupational pension scheme; or (5) receives basic social security payments. In turn, F. Denton and B. Spencer [124] distinguish eight ways in which retirement is defined in the literature. These are: (1) not participating in the labor force; (2) reducing the number of hours worked or the sum of earnings; (3) working the number of hours or receiving earnings below a certain limit value; (4) receiving income from retirement; (5) leaving work at the main employer; (6) changing occupation or employment later in life; (7) subjective declaration of the person concerned; (8) various combinations of the above-mentioned criteria. An attempt to generalize the presented ways of defining the concept of retirement leads to the distinction of four basic criteria that can be taken into account when defining it:

- According to the type of income criterion, a pensioner is a person who receives a retirement benefit.
- According to the event criterion, a pensioner is a person who has partially or completely reduced the supply of his work or changed its nature.
- According to the age criterion, the population of pensioners includes people who have exceeded a certain metric age.
- According to the declarative criterion, a pensioner is a person who defines himself as a pensioner.

In absolute terms, it is not possible to clearly indicate which of the indicated criteria for defining retirement is more appropriate and justified. On theoretical grounds, attempts to formulate a universal definition of retirement—especially if it is to cover both the economic and psychological aspects of the process—are therefore doomed to failure. Conducting empirical research, however, requires a specific moment when retirement occurs. Therefore, when attempting to operationalize the concept of retirement for purposes of the research discussed below, it was considered that this event would be treated as occurring simultaneously: the final cessation of professional activity and the start of receiving pension benefits. The justification for the use of such an approach—necessarily simplified—is the adopted research method, within which the planned retirement age declared by the participants of the survey is considered.

### 3.3. Research Procedure and Research Questionnaire

The research tool for the verification of hypotheses (H.1–H.3) was an original research questionnaire. This questionnaire was not only a simple instrument for surveying the opinions and attitudes of respondents, but an extensive tool aimed at the implementation of experimental research. Therefore, its development was a complex undertaking involving several stages: (1) preparation of a preliminary version of the questionnaire; (2) expert consultations with specialists in the field of retirement economics, behavioral finance and psychology (see acknowledgments); (3) development of a digital version of the research tool (CAWI); (4) pilot study and cognitive interviews conducted on a non-random sample of respondents; (5) preparation of the final version of the questionnaire. 

In the final version, the research questionnaire, in addition to filtering and statistical questions, contained 19 questions on various conditions of starting retirement. Of these questions, this study included those that related to the framing effect. The hypotheses were tested by analyzing the structure of answers to selected questions and analyzing the relationship between answers to different questions contained in the research questionnaire. The study used statistical tools such as classical and positional measures, nonparametric Kruskal–Wallis and U Mann–Whitney tests as well as statistical modeling tools using multiple logistic regression models.

In the designed study, two specific experimental measures were used to investigate the impact of the framework effect on decisions regarding the planned retirement age: a description of a hypothetical pension system and a multi-variant vignette question.

At the beginning of the survey, respondents were familiarized with the description of a hypothetical pension system in which there is freedom to decide on the choice of retirement age:

“*Experts are currently discussing the assumptions of a completely new proposal for a pension system. This new pension system will give you the opportunity to decide freely regarding the moment of leaving your job and starting to receive your pension. In the proposed pension system, if you pay contributions to the pension system throughout the entire period of work and retire at the standard retirement age (the age is assumed to be {65 years}), the amount of the pension will be about {40%} of your last gross salary. Retiring one year before the standard retirement age will mean about 10% lower retirement benefits for the rest of your life for each year of shorter work. Similarly, retiring one year after reaching the standard retirement age will entail about 10% higher pension for each additional year of work.*”

The use of a description of a hypothetical pension system in the study was aimed at separating respondents from the considerations embedded in the current structure of the pension system. This procedure was therefore intended to enable the analysis of real preferences regarding the planned retirement age without potentially burdening the response with current systemic solutions. A similar treatment has also been used in other studies on the behavioral determinants of retirement decisions [87,93].

The question to verify the impact of the framing effect on the planned retirement age had the formula of an experimental vignette question. In questions of this type, the subject is asked to indicate what he/she did in the place of the person presented in the hypothetical description of a specific situation. The form of vignette questions is known and successfully used in research on retirement attitudes and behaviors [93,125]. Compared to direct questions, the biggest advantage of vignette questions is the ability to control selected variables and manipulate them by asking subjects different versions of the same question. Other advantages of vignette questions over direct questions include: (1) greater realism; (2) ensuring standardized stimulants for all respondents, which increases the internal relevance of the research, the reliability of the measurement and the ease of replication; (3) increasing the accuracy of questions, by directing respondent attention to specific aspects of the research problem; (4) limiting the tendency to give affirmative answers (yea-saying bias) and answers considered desirable; (5) increasing the involvement of respondents and polarizing the problem posed [126].

The use of the vignette question in this study was aimed at an isolated analysis of the impact of modified variables on respondent preferences regarding the planned retirement age. According to the general idea of vignette questions, respondents confronted with the description of the situation of fictitious persons should take into account only those variables that are presented in the content of the question. In the process of making decisions on behalf of a fictitious person, respondents should not take into account other variables that, in fact, if a similar question were asked in a direct form, would affect their answers.

In the survey, respondents were randomly presented with one of the five variants of the question (see Table 2 and Table A1 in Appendix A). Individual variants were the same in terms of content, and they were differentiated only by the values of the parameters presented in them: the age of retirement and the amount of the benefit (in the content presented above, these variables are included in curly brackets. To avoid gender bias the participating men were shown variants of the question with a male name and corresponding pronouns, and women with a female name and pronouns. The values of both these parameters were selected so that the individual variants differed only in the way of formulating the decision-making problem. In terms of the relationship between the retirement age and the amount of the benefit, all variants were identical. The base variant was set as a retirement benefit at the age of 65 at the level of PLN 2000. According to the assumptions of the hypothetical pension system, each shortening/extension of the retirement age by one year meant a decrease/increase in this benefit by approximately 10%.

The first column of Table 2 includes the concept of framing direction. The influence of framing direction on the decision-making process is well-recognized in the literature. The potential relationship of loss and gain framing with retirement decisions is also discussed in Section 2.3 “Framing Effect and the Decision to Retire”. Therefore, there is no need to explain it here in detail. The concept of narrow and broad framing range presented in the second column of Table 2, however, requires additional explanation. When designing the research, it was assumed that the framing effect may depend on specific values presented to the respondents as part of the decision-making problem. According to one of the research suppositions (see hypothesis H.3), if the formulation of the decision-making problem differs only slightly from the neutral variant, the impact of the framing effect will be weaker than in the case of formulating the same decision-making problem in a more extreme version. Hence, bearing in mind the above supposition, “narrow” and “broad” variants of framing were formulated. In the “narrow” variants, the difference between the subsequent retirement years presented in the vignette question was 2 years (the respondent could choose any retirement age, but the presented framework was in the range of 63–67 years). In turn, in the “broad” variants the difference was 4 years (the framework ranged between 61 and 69 years).

The research structure behind the formulation of variants of the question assumed that people confronted with different variants of the question would give different answers. If this presumption was correct, it would be a premise in favor of the truth of hypothesis H.1. Another assumption was that respondents confronted with variants emphasizing losses (V4, V5) would indicate on average a higher retirement age than people who have read the options emphasizing profits (V2, V3). To show the truth of this conjecture would be a confirmation of the validity of hypothesis H.2. Similarly, proof of the validity of the H.3 hypothesis would be to show that, compared to those who were presented with a narrower framework for the decision-making problem (V2 and V4 variants), those who were presented with a broad framework declared a higher planned retirement age (V3 and V5).

### 3.4. Scope of the Research

The subjective scope of the research included participants of the general pension system in Poland who have not yet acquired pension rights: women aged 40–59 and men aged 45–64. The introduction of the upper age limit was aimed at excluding from the survey people who have already reached the statutory retirement age. Currently, this age in Poland is set at 60 years for women and 65 years for men. The lower age limit was introduced to include in the study only those people who could realistically start thinking about their future in retirement since, according to T. Beehr’s model of retirement [127], these are people who have found themselves at least in the first phase of the retirement process (imagining a future life in retirement). The beginning of this phase was subjectively set at 20 years before reaching the current statutory retirement age.

The difference in the statutory retirement age of women and men in Poland is a justification for a different determination of age ranges for respondents of both genders in surveys based on the planned retirement age. At the same time, this fact poses some methodological problems, as the group of respondents is not homogeneous in terms of age. For this reason, in order to identify the possible consequences of this circumstance, the analyses presented in the next section were carried out separately for the entire population and separately for women and men. In addition, in each of the analyzed models, the gender and age of respondents were taken as one of the control variables.

### 3.5. Data

The empirical study using the research questionnaire was carried out using the CAWI (Computer-Assisted Web Interview) method in February 2021 among randomly selected participants of the Reaktor Opinii research panel created by the Pollster Research Institute. The panel has been operating since 2014. Currently, it is attended by 176.7 thousand panelists who have undergone a multi-stage registration process. In order to ensure the reliability of the research data received, the panel uses an automatic system that monitors suspicious results, consistency of responses and the time of their provision by individual respondents. In addition, research projects carried out on the panel are supervised by a team of analysts who keep track of the progress in the implementation of individual studies and verify their correctness Pollster Research Institute (www.pollster.pl) (accessed on 15 December 2021); Reaktor Opinii (https://reaktoropinii.pl/) (accessed on 15 December 2021). From the point of view of the reliability of the research and the reliability of the results obtained, it is important to state that the participants of the panel receive remuneration for completing the surveys. 

Despite the efforts made to randomly select and randomize the subjects as extensively as possible, the method of selecting the research sample meant that it was not formally fully representative. The sample selection burden was related to two issues: (1) the profile of the people participating in the research panel (by definition, these were people using a computer); (2) the underweight of the characteristics of respondents who did not qualify for the survey as a result of filtering (e.g., people not covered by the general pension scheme). It should be emphasized, however, that the conducted study was a research experiment, which, according to the methodology of behavioral economics, does not require the fulfillment of rigorous assumptions as to the representativeness of the research sample.

In total, 1114 respondents took part in the survey, of which 1079 met the full eligibility criteria for the analyzed sample. Basic information on the structure of the test sample is provided in Table 3.

## 4. Results

**Figure 1:**
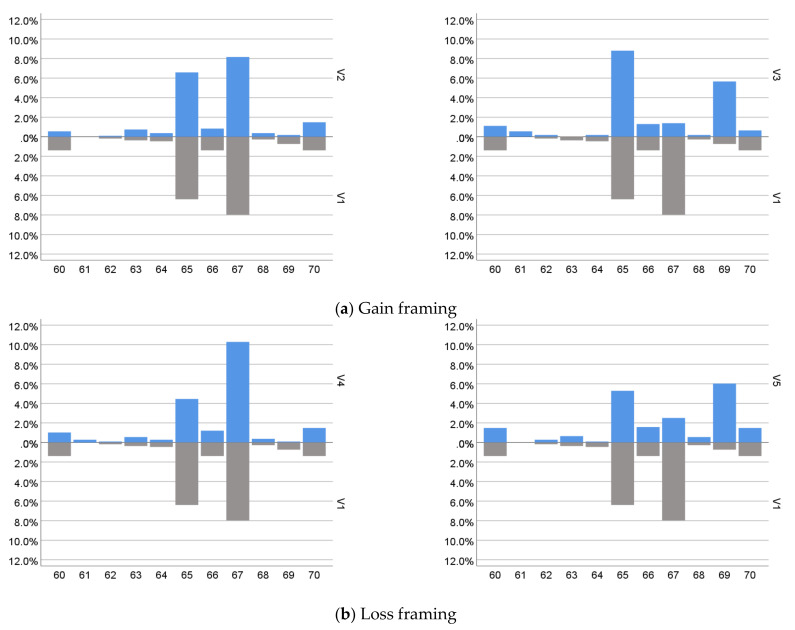
The impact of the framing effect on the retirement age. (**a**) Gain framing; (**b**) Loss framing.

The distributions of answers given to the five variants of the question of planned retirement age are shown in Figure 1. The graphs on it illustrate the comparison of the answers given by respondents in the neutral variant (V1) with subsequent variants taking into account the gain framing (V2 and V3) and loss framing (V4 and V5).

A preliminary comparison regarding the distribution of responses to different variants of the vignette question suggests that the formulation of the decision-making problem did indeed affect the planned retirement age of the respondents (see Table A1 in Appendix A). Based on the analysis of the frequency of responses, the following observations can be formulated:

- Compared to the groups presented with a neutral variant of the question (V1), respondents who read the questions taking into account one of the framing options, indicated on average a 3 month higher planned retirement age.
- Comparison of answers to questions with loss framing (V2 and V3) with questions with gain framing (V4 and V5) indicates that highlighting losses in the question leads to a slightly higher planned retirement age (66 years and 4 months and 66 years and 1 month, respectively).
- Comparing the answers to questions taking into account narrow (V2 and V4) and wide (V3 and V5) framing did not give unambiguous results. On the one hand, both variants of gain framing have produced similar results: extending the planned retirement age by about 2 months. On the other hand, the difference between the loss framing options was significant: the narrow framework led to an extension of the planned retirement age by 2 months, while the wide one was by 7 months.

Analyses of the distribution of responses given by women and men indicate the existence of some gender differences (see Appendix A). In particular, it was noted that the average planned retirement age of women (65.9 years) was 7 months lower than that of men (66.5 years). In addition, an analysis of the responses suggests that women were more affected by the framing effect. While the average effect of all framing variants among women was equal to 5 months, among men it was 1 month. Women were also more susceptible to gain framing (among men this effect did not occur, among women it was 4 months) and loss framing (the effect among men was 2 months, and among women it was 7 months).

When analyzing the impact of the framing effect, one should also note a certain anomaly observed in the group of men. Contrary to expectations, the average planned retirement age in the V3 and V4 variants turned out to be lower by 2 months and 1 month, respectively, than in the natural variant (V1). A potential explanation for this situation is the strong anchoring of men at the statutory retirement age of 65. This topic will be discussed in more detail in the next section.

Previous analyses have been based on descriptive statistical tools and have referred only to the characteristics of the research sample. In order to deepen the analyses and verify the significance of the observations made in relation to the entire population, the nonparametric Kruskal–Wallis test was used (H_0_: the compared distributions have the same distributives). The obtained values of the test statistics indicate that the differences in the answers given by people who were familiar with the individual variants of the questions were statistically significant (H(4) = 10.91; *p* = 0.028). In order to determine which of the compared distributions differed from each other in a statistically significant way, post hoc comparisons were carried out using Dunn’s multiple comparison test [128]. (see Table 4) 

The Bonferroni-corrected *p*-value [129] indicates that statistically significant (α < 0.1) differences in the compared distributions were observed when comparing the neutral variant (V1) with loss-oriented variants with a baseline age of 69 years (V5). In addition, significant differences were also observed when comparing both variants presenting a broader framework of the decision-making problem (V3 with V5). These results suggest that of the framing variants compared, the V5 variant had a statistically significant impact on respondent responses. Kruskal–Wallis tests conducted separately in the groups of women and men were ordered (with a significance level of α > 0.05) to adopt zero hypotheses about the equality of the compared distributions (for women H(4) = 8.791; *p* = 0.067; for men H(4) = 4.286; *p* = 0.369). When interpreting this result, however, one should take into account the smaller number of these sub-samples (n equal to 569 and 510, respectively).

In the next stage of the study, the logistic regression method was used. This method allows the examination of the influence of explanatory (independent) variables on the probability of occurrence of the analyzed event. Since logistic regression is one of the more advanced statistical methods and is not widely used in research on retirement decisions, it requires a short comment here (however, in some studies on individual retirement decisions, logistic regression models have been applied. See e.g., [130,131,132,133]. The logistic regression method is characterized in more detail in statistical and econometric studies like [129,134,135,136,137,138].

The general form of the logistic regression model is defined by a relationship [129]:

(1) $$P\left(Y\right)=\frac{1}{1+e^{-\left(\beta_{0}+\beta_{1}X_{1}+\beta_{2}X_{2}+\cdots +\beta_{n}X_{n}+\varepsilon_{i}\right)}},$$

where: *P(Y)*—probability of event Y, $X_{i}$—*i*-th explanatory variable, $\beta_{i}$—coefficient (weight) of the *i*-th explanatory variable, $\beta_{0}$—constant.

The most important feature of logistic regression is that the explained variable in it is dichotomous (takes the values 0 or 1). Explanatory variables, on the other hand, can be measured on a quantitative scale, and after being recoded into zero-one variables, also on a nominal or ordinal scale. In order to estimate the parameters of the logistic regression model—unlike classical linear regression, which is based on the method of least squares—the maximum likelihood method is used. Hence, the assumptions of logistic regression models are less restrictive than the assumptions of linear regression models. In particular, logistic regression allows for the heterogeneity of variance and the absence of a normal distribution of explanatory variables and residues. Among the conditions necessary for the application of logistic regression are the lack of collinearity of variables and the appropriate group size [139]. In general, it is assumed that the sample size n must satisfy the condition *n* > 10 (*k* + 1), where k is the number of parameters in the regression model.

To determine statistical significance for each of the independent variables in the logistic regression model, Wald test is used. The test statistics in the Wald test are calculated on the basis of the value of the estimated parameters and their standard errors. To evaluate the overall model significance the omnibus tests of model coefficients that is based on the chi-square statistic may be used. The quality of a logistic regression model is assessed using the pseudo R2 coefficients (Nagelkerke R2 or Cox and Snell R2). These coefficients are adjusted equivalents of the multiple determination coefficient R2 calculated for standard linear regression models. Another way of assessment of the quality of the regression models is the Hosmer–Lemeshow test. It tests the null hypothesis that predictions made by the model fit perfectly with observed group memberships. In this test, a chi-square statistic is computed comparing the observed frequencies with those expected under the linear model. Importantly, a nonsignificant chi-square indicates that the data fit the model well. Therefore, rejecting the null hypothesis in this test is desirable [139]. 

After building a logit model, to assess its quality, the ex-post analysis can be performed on the basis of classification tables. In this analysis, an ex-post forecast of the value of the dependent variable for each observation was calculated. An ex-post analysis is used to calculate values such as accuracy, sensitivity and specificity of the model. In addition, ROC curves and the AUC measure are also useful methods for assessing the quality of linear regression models.

In the interpretation of the results of estimating the parameters of logistic regression, the key is the antilogarithm $exp\left(\beta \right)$ called the odds ratio (*OR*).

For any variable *X* it is given by the following formula [134]:

(2) $$OR\left(X\right)=\frac{P\left(X\right)}{1-P\left(X\right)}$$

It indicates the ratio of the odds of occurrence of a given event (the explanatory variable taking the value of 1) in relation to the reference group. For continuous and dichotomous independent variables, one odds ratio is calculated. If the independent variable has more values, the number of possible odds ratios is as many as the categories of the analyzed variable. The odds ratio has a similar interpretation to the β coefficient in the standard linear regression model.

On the basis of its value, it is assessed whether and to what extent an increase in the value of a given predictor causes a decrease or increase in the probability of the occurrence of the analyzed phenomenon measured by the dependent variable. Wherein:

- $Exp\left(\beta \right)>1$ means that, in comparison with the reference value, the given value of the independent variable increases the odds of the occurrence of the analyzed phenomenon;
- $Exp\left(\beta \right)<1$ means that, in comparison with the reference value, the given value of the independent variable decreases the odds of the occurrence of the analyzed phenomenon;
- $Exp\left(\beta \right)\approx 1$ means that, in comparison with the reference value, the given value of the independent variable does not change the odds of the occurrence of the analyzed phenomenon.

In the case of continuous variables, the odds ratio shows the change in the logarithm of the odds ratio when the value of the independent variable changes by 1 [129,140].

In the presented studies, two separate analyses were carried out using logistic regression to check how the probability of the planned retirement age is affected by the framing effect. In these analyses, the probability of planned retirement at the age of:

- Over 65 years of age—analysis 1,
- Over 67 years—analysis 2,

was examined. Each analysis estimated three regression models that included the total population (1P and 2P models), women (1W and 2W models) and men (1M and 2M models), respectively.

With regard to the conducted research, the initial model of logistic regression takes the following form:

(3) $$P\left(Y\right)=\frac{1}{1+e^{-\left(\beta_{0}+\beta_{1}X_{1}+\beta_{2}X_{2}+\beta_{3}3+\beta_{3}X_{3}+\beta_{4}X_{4}+\beta_{4}X_{4}+\beta_{5}X_{5}+\beta_{6}X_{6}+\beta_{7}X_{7}+\beta_{8}X_{8}\right)}},$$

where: *P(Y)*—probability of retiring over the indicated age (65 in analysis 1 and 67 in analysis 2), $X_{1}$—framing effect; $X_{2}$—gender; $X_{3}$—age; $X_{3}$—education; $X_{4}$—financial situation; $X_{5}$—health condition; $X_{6}$—marital status; $X_{7}$—number of children; $X_{8}$—type of job; $\beta_{1},\;\beta_{2},\beta_{3},\beta_{4},\beta_{5},\beta_{6},\beta_{7},\beta_{8}$—coefficients of the respective explanatory variables; $\beta_{0}$—constant (intercept).

In the estimated models the explanatory variable (Y) was the planned age of retirement. This variable was dichotomized by assigning it 1 when its original value was equal to or higher to the age of the subject in a given analysis and 0 in the opposite case. As explanatory variables, a variant of the vignette question verifying the impact of the framing effect $\left(X_{1}\right)$ and a set of variables representing factors taken into account in neoclassical pension models, which in the study were control variables, were adopted ($X_{2},X_{3}\ldots X_{8}$). These variables included: age, education, financial situation, health status, marital status, number of children and nature of work performed. Importantly, the financial situation and health status were assessed by respondents subjectively (on the Likert scale), and not using objective but often unrealistic measures such as current gross income, amount of retirement savings or the number of days of incapacity for work per year.

After building a formal logit model (Equation (3)), its structural parameters were estimated. When analyzing the presented results, it was noticed that not all variables included in the model had a significant impact on the probability of retiring over the indicated age. Therefore, in the next step, to fit the model and eliminate irrelevant variables, the stepwise regression was used. Taking into account the method of selecting explanatory variables for the study (predefined set of variables), the Wald’s backward stepwise selection method was used [129]. In this method, checking whether a given variable is subject to removal is based on the probability of Wald’s statistic defined by the formula:

(4) $$\mathrm{Wald}\left(Z\right)=\frac{\beta_{i}}{SE_{\beta }}$$

Subsequent variables are removed from the model if the value of the test probability of the corresponding Z-statistic is greater than the adopted significance level (in the study the significance level of 0.1 was adopted). Regression models which were built using backward selection are presented in Table 5 (whole population) and in Appendix B (models for women and men).

When interpreting the results obtained in the performed analyses, it should be noted that the estimated regression models make it possible to explain decisions regarding the planned retirement age to a limited extent (see the respective values of Cox and Snell R2 and Nagelkerke R2). Nevertheless, the obtained results confirm that each of the estimated models is statistically significant (in each model the omnibus test’s *p*-value < 0.001). In addition, the results of the Hosmer–Lemeshow tests (*p*-value > 0.05) indicate a good fit of these models and justify the correctness of the inference concerning the influence of selected variables on the probability of retirement above a given age. Additional analyses of accuracy, sensitivity and specificity of the estimated models are presented in Appendix C. They show that the accuracy of the models in analysis 1 ranges from 62.7% to 65.7%, and in analysis 2 from 75.3% to 83.8%. The Appendix C also contains graphs of the ROC curves drawn for each of the models and the values of respective AUC measures (for the models in the first analysis, AUC ranges from 0.661 to 0.664, and for the models in the analysis 2, from 0.716 to 0.811).

The results of the regression analyses indicate that in both estimated models (1P and 2), the framing effect was a variable that statistically significantly affected the probability of planned retirement above a certain age (Wald test *p*-value for the overall variable “framing effect” equal to 0.001 and 0.000, respectively). The estimated parameters of the 2P model show that respondent decisions were significantly influenced by a broader framework of decision-making problems. In this model, compared to the neutral variant (V1), people who were familiar with the question variant with a framing emphasizing profits with a starting age of 61 years (V3) had 3.89 greater odds to indicate the retirement age above 67 years. Similar odds for those presented with a variant of the question with a framing emphasizing losses with the starting age of 69 years (V5) increased 5.26 times. At the same time, in model 2P both loss and gain framing in narrow frameworks (V2, V4) were statistically insignificant.

Significance tests for dummy variables’ coefficients in model 1P gave inconclusive results. On the one hand, the V2 and V5 variants were statistically insignificant. On the other hand, a significant impact (*p* < 0.1) of the variant (V4) was noted: it increased the chances of planned retirement over 65 years by 1.45 times. The impact of the V3 variant was also significant. Contrary to expectations, however, this option reduced the analyzed chances by 39%. As in the case of the average planned retirement age, a potential explanation for this phenomenon may be a strong anchoring of the respondents at the statutory retirement age limit (65 years), which is discussed in the next section.

The parameters of the regression models estimated separately for women and men (see Appendix B) confirmed the observations on the significant impact of framing options with a broad framework on the probability of indicating and the retirement age above 67 years. They also allowed for the conclusion that the observed framing effect had a greater impact on women. This is evidenced by both the statistical significance of the estimated odds ratios in particular models and the numerical values of these ratios. For example, the V3 variant increased the chances of women to indicate retirement age over 67 by 8.35 times, and men by 2.41 times. In turn, the V5 variant increased the chances of women to indicate the age over 67 years as much as 14.27 times, while among men it was only 2.47 times.

It should also be noted that, as in the case of model 1P for the general population, the models of indicating the retirement age above 65 years estimated separately for women (model 1W) and men (model 1M) do not allow one to draw unequivocal conclusions about the impact of the framing effect. On the one hand, the V4 variant had a statistically significant influence on women, increasing the chances of planned retirement above 65 by 76%. On the other hand, in the case of men, it turned out that the V3 variant had a significant impact, but it caused a 42% decrease in the analyzed chances. Once again, the reason for such results may be the anchoring of the statutory retirement age of 65 which, due to the architecture of the Polish pension system, is particularly strong among men.

In addition to the conclusions regarding the impact of the framing effect on decisions on the planned retirement age, the analyses allowed one to make some observations on the impact of classical factors considered in standard pension models:

- Both in model 1P and 2P, the variable significantly influencing the planned retirement age was gender. If the respondent was male, the odds of indicating the retirement age above 65 years increased by 85%, and the odds of indicating the retirement age above 67 years increased by 125%.
- A factor influencing the planned retirement age was the age of the respondent. In each model (both the total population and for women and men studied separately), the values of odds ratios indicated that an increase in the age of the subject by one year caused a decrease in the odds of retirement above the age taken into account in a given model (this value fluctuated in the range of 4–5%). The older the person was (and the closer they were to actual retirement), the less willing they were to prolong their professional activity. As potential explanations for this relationship, one can point to changing external conditions, changes in the mentality of the subjects as well as the phenomenon of hyperbolic discounting discussed in behavioral economics [141,142]. However, a full explanation of the identified relationship requires further research.
- Better education was associated with greater chances of indicating a higher retirement age. The effect of education was stronger in the case of men. For example, compared to men with primary education, men with higher education had 3.42 times higher odds to indicate the planned retirement age over 65, and 3.88 times higher odds to indicate the planned retirement age above 67. The respective values for women were 2.51 and 2.03. This observation proves the key role of broadly understood education in stimulating the extension of professional activity.

Among other control variables which had a significant impact on the probability of a planned retirement above 65 was marital status (in models 1P and 1W). In the case of men, the significant variables were also number of children and health status. The former had an impact on planned retirement age in model 1M. Surprisingly, having more children reduced the probability of indicating the planned retirement age above 65. The latter, to some extent, affected the chances of retirement in the model 2M.

The analyses carried out did not show that the financial situation, which is one of the key factors considered in neoclassical pension models, had a significant impact on the planned decision to retire. The research also did not prove the existence of any significant relationships between the planned retirement age and the nature of the work performed.

## 5. Discussion

Summing up the completed research, one can state that they support the adoption of the H.1 hypothesis. Both the comparison of response distributions and the estimated regression models showed that, compared to the neutral option, the variants with framing led to an extension of the planned retirement age. Therefore, it was considered that the problem of decision-making regarding retirement affects the extension of the planned retirement age. This conclusion is therefore in line with the prevailing view in the literature [109,110,113,122]. Analyzing the obtained results, however, it is necessary to point out that the impact of individual framing variants was differentiated. This observation is confirmed by the results of the verification of the other two research hypotheses discussed further.

With regard to the H.2 hypothesis, it was found that the result obtained provides sufficient grounds for recognizing its veracity. An analysis of the response rate in the sample showed that the average planned retirement age indicated by those presented with loss framing is higher than the average age indicated by those presented with a gain framing. This observation is also confirmed by analyses of response distributions. In particular, post-hoc tests have shown that the planned retirement age distributions are significantly different for the selected loss-oriented and gain-oriented options. Partial confirmations of the H.2 hypothesis are also the estimated values of parameters of logistic regression models, which suggest that the probability of retirement above a certain age is more strongly influenced by loss framing. The conclusions drawn are partly consistent with the earlier results of D. Fetherstonhaugh and L. Ross [109] who argued that the framework emphasizing losses to a greater extent than the framework emphasizing profits affects the extension of the planned retirement age. At the same time, however, these conclusions are not consistent with the claims of J. Liebman and E. Luttmer [110], who stated that the framework emphasizing profits has a greater impact on the extension of labor force participation than the framing emphasizing losses.

In the case of H.3, according to which the wider framing of the decision-making problem (more than narrow framing) affects the extension of the planned retirement age, it was found that the results obtained were inconclusive. In the case of gain framing, no significant relationships were identified. On the other hand, in the case of loss framing, comparing the frequency of answers to questions taking into account broad and narrow framing suggested that a wider definition of the frame of the decision-making problem resulted in a greater extension of the planned retirement age. However, this assumption was not confirmed by the analysis of distributions and post-hoc tests carried out. The parameters of the estimated regression models indicated that, depending on the explanatory variable, the parameter responsible for the broader or narrower framing of the decision problem emphasizing the losses was important. Hence, in the end, the H.3 hypothesis was left unresolved, while indicating that its verification is a desirable direction for further research.

Regardless of the research hypotheses verified, the study noted that the framing effect is more pronounced among women than men. This is evidenced by both the distributions of the responses received and the estimated parameters of regression models and their significance. This observation therefore contradicts the earlier results of research by J. Brown, A. Kapteyn and O.S. Mitchell [113], who, on the basis of a study conducted in the USA, came to the conclusion that men are more susceptible to the effects of the framing effect. This observation prompts reflection on what may be the reason for the greater impact of manipulation of the way the problem is presented on the decisions made by women. One of the potential explanations for this phenomenon may be the impact that the exemplary values of the retirement age given in individual variants of the question had on the answers given by women. It can also be assumed that the lower susceptibility of men to manipulation may be related to the further discussed anchoring effect at the age of 65. This age appeared in each variant of the analyzed question and could place some burden on the answers provided. However, it was not possible to verify the above assumptions on the basis of the analyzed data set.

In addition to the conclusions on the effects of the framing effect, the research also made some observations on the impact of other behavioral determinants on decisions about planned retirement. Figure 1 shows that the shapes of the response distributions are far from the shape of the normal distribution (that would be expected if the preferences for retirement age were in no way burdened by behavioral inclinations. This observation also applies to the aggregate distribution for all variants of the question, and is confirmed by the results of the Kolmogorov–Smirnov normality tests with the Lilliefors correction (total population: Z(1079) = 0.201; *p* < 0.001; for women Z(569) = 0.220; *p* <0.001; for men Z(510) = 0.177; *p* < 0.001). It seems that it is no coincidence that the most frequently indicated retirement age was 65 years (31.5% of total responses) and 67 years (30.3% of total responses), and in V3 and V5 variants also 69 years (12.7% of total responses, respectively).

The relatively frequent indication of the retirement age of 65 can be explained by the anchoring effect. The value of 65 years corresponds to the limits of the statutory retirement age for men, which have been in force in Poland since the 1950s. It is so deeply rooted in the consciousness of respondents that it acts as a retirement age anchor for respondents [87]. This observation is consistent with the results of studies carried out by N. Vermeer M. van Rooij and D. van Vuuren [93], which show that the Dutch tend to prefer the retirement age of 65, which for many years was the statutory retirement age in the Netherlands. This result is also confirmed by C. Brown [86], who noted the anchoring of the USA population at the retirement age of 62 and 65 years.

The answer of the 67 and 69 years may have been influenced by the suggestion contained in the content of the question. Respondents who were presented with a variant of the question containing in the description a reference to the retirement age of 67 years (V1, V2, V4) were much more likely than other people to indicate this retirement age. Similarly, respondents who were presented with options taking into account the possibility of retirement at the age of 69 (V3 and V5) were much more likely to choose this value of the retirement age in response. At the same time, the answers between the values of the retirement age included in the individual variants of the question (66 and 68 years) were indicated much less frequently. These observations are therefore an argument in favor of the thesis put forward in the literature concerning the impact of default options on the retirement age [87,92].

When discussing the conducted research, it should be noted that, in addition to the methodological and empirical contribution (development and application of methods for assessing the impact of the framework effect on retirement decisions), it also has application values. The obtained results may be useful in creating pension policy and labor market policy. For example, the intentional use of the proven impact of the framing effect on pension decisions can be used to stimulate longer working lives. Behavioral intervention could consist of periodically providing insured persons with information on the amount of the benefit in the event of a delay in retirement, with the suggestion that earlier (i.e., at general retirement age) claiming of this benefit will reduce the amount of the benefit by a certain amount. It can be presumed that, just as the automatic enrolment mechanism translates into the level of participation in voluntary pension plans [88,107], a change in the architecture of the decision to retire will affect the postponement of the decision to end professional activity.

Despite the interesting findings, the research carried out has some limitations. A limitation of a methodological nature is the subject of research. The study assumes that “retirement” is determined by the respondents declaratively. Therefore, the research does not take into account the different possibilities of defining this phenomenon, in particular the fact that retirement does not have to be the same as the end of professional activity. In addition, the study is based on the concept of a planned retirement age. Despite the many advantages of such a solution, when interpreting the results obtained, it should be borne in mind that with the change of personal conditions and external circumstances, pension plans may be subject to modifications. Another limitation is the specificity and quality of the research sample. First, the research was limited to participants in the universal Polish pension system. Secondly, despite the author’s best efforts, the method of sample selection could nevertheless have some impact on the results. Some limitations are also related to the results of quantitative analyses: the statistical procedures used did not always definitively determine whether the analyzed relationships were causal or only correlative. The presented interpretation of the results is therefore the result of the synthesis of the obtained empirical results and theoretical investigations. Another point is that the estimated logit models were not always well suited to the data, which resulted in a limited possibility of inferring from them and drawing unequivocal conclusions.

The conducted research may form the basis for further, in-depth analyses. In particular, it is desirable to examine the operation of the default options of the retirement age and their relationship to the framing effect. A promising direction of research is also the verification of the impact of other behavioral conditions listed in the literature review on decisions about retirement. It is also worth devoting further research to checking the degree of succumbing to the effect of framing by people with different socio-demographic and psychographic characteristics. A possible extension of the presented research is also to carry out similar analyses based on larger research samples, in other countries, as well as in the international dimension.

## 6. Conclusions

The presented study verified how the formulation of the decision-making problem (the framing effect) affects decisions regarding retirement. On the basis of experimental questions included in a survey conducted among non-retired participants of the Polish pension system, it was proven that the framing effect affects the extension of the planned retirement age. The conducted research allows one to conclude that loss framing has a greater impact on respondent decisions than gain framing. At the same time, no evidence was found that broad framing rather than narrow framing extends the planned retirement age. An important application conclusion from the research is the finding of the possibility of the intentional use of the framework effect by decision-makers in order to stimulate the extension of the professional activity of older people.

## Figures and Tables

**Table 1:** Behavioral Factors Affecting Retirement Decisions.

Behavioral Factor	Characteristics
Framing effect	In the case of decisions on retirement, the framing effect is the impact of how the decision-making situation is presented on the choices of decision-makers regarding their actual or planned (declared) retirement age.
Default option	A standard choice for which no additional action is required. It is a decision that requires the least analysis and intellectual effort. The person deciding on the default option is satisfied with the consequences of this option and is not looking for the optimal solution, in terms of cost–benefit analysis. In the context of the decision to retire, a typical default option is a general retirement age.
Anchoring effect	Influencing pension decisions by values that are deeply rooted in the consciousness of policymakers and are important for retirement (age anchors) [87]. Such values are legal figures that persist for many years, such as the minimum or general retirement age.
Impact of social norms and social environment	The influence of other people’s opinions, beliefs and experiences on the decisions made. It may be direct or indirect: direct influence consists of the influence of the immediate environment (family, friends, co-workers, superiors). Within this channel of influence, individuals learn from each other through discussions or by drawing conclusions from actions taken by others; indirect influence consists of the influence of abstract norms that are common in larger social groups. Individuals try to act in such a way that, after the end of professional activity, they maintain a similar level of consumption to that which is common in their social group [114].
Hyperbolic discounting	Perceiving future benefits well below their real value and overestimating the value of benefits offered immediately. This phenomenon explains why people who initially planned to retire later actually retire near the statutory retirement age.
Planning fallacy	Erroneous prediction of future events as a result of the unrealistic (excessively optimistic) construction of mental scenarios that the individual creates to predict the future. When considering retirement, people analyze only the best scenarios (they do not take into account negative random events such as illness or death of a partner), which makes them willing to retire earlier and accept lower retirement benefits.
Affective forecasting	People’s tendency to imagine that a given event in the future will be better (or worse) than it later turns out. M. Knoll transferred onto pension economics the concept of affective forecasting [96]. She argued that this phenomenon leads individuals to prefer early retirement, as people tend to imagine that retirement will bring them more satisfaction than it actually does.

Source: own study based on [85].

**Table 2:** Variants of Framing the Decision-Making Problem.

Framing Direction	Framework Range	Question Variant Code	Retirement Age Presented in the Question			Number of Respondents	
			Initial	2nd	3rd	Women	Men
Neutral	-	V1	65	63	67	118	104
Gain	Narrow	V2	63	65	67	111	98
Gain	Broad	V3	61	65	69	116	100
Loss	Narrow	V4	67	65	63	110	107
Loss	Broad	V5	69	65	61	114	101
Total:						1079	

**Table 3:** Structure of the Research Sample.

Characteristics	Number of Responses	Share (%)
Total	1079	100
Sex		
– Woman	569	52.7
– Man	510	47.3
Age		
– 40–49	159	14.7
– 45–49	275	25.5
– 50–54	281	26.0
– 55–59	242	22.4
– 60–64	122	11.3
Education		
– Basic	268	24.8
– Average	466	43.2
– Higher	345	32.0
Type of job		
– professionally inactive	110	10.2
– intellectual, office, administrative work	296	27.4
– manual labor	260	24.1
– profession requiring contact with people, team management	218	20.2
– highly specialized profession	88	8.2
– pensioner (disability)	107	9.9
Marital status		
– without a partner	271	25.1
– has a partner	808	74.9
Number of children		
– lack	205	19.0
– one	286	26.5
– two	417	38.6
– three or more	171	15.8
Domicile		
– village	393	36.4
– city up to 50,000 inhabitants	250	23.2
– city from 50,001 to 200,000 inhabitants	219	20.3
– city over 200,001 inhabitants	217	20.1

**Table 4:** Post Hoc Comparisons for Different Framing Variants.

Compared Variants	V1-V2	V1-V3	V1-V4	V1-V5	V2-V3	V2-V4	V2-V5	V3-V4	V3-V5	V4-V5
Z statistics	−0.303	−0.217	−1.288	−2.836	0.087	−0.968	−2.493	−1.063	−2.601	−1.542
*p*-value	0.762	0.828	0.198	0.005	0.931	0.333	0.013	0.288	0.009	0.123
Bonferroni-corrected *p*-value	1.000	1.000	1.000	0.046	1.000	1.000	0.127	1.000	0.093	1.00

**Table 5:** Logistic Regression Models of Planned Retirement Age (Whole Population).

Variable	Model 1P					Model 2P				
	(>65 Years)					(>67 Years)				
	β	SE β	OR	Sig	95% CI for OR	Β	SE β	OR	Sig	95% CI for OR
Framing effect (ref.: V1 (neutral))				***					***	
– V2 (gain, narrow)	−0.04	0.20	0.96		0.64–1.42	−0.14	0.31	0.87		0.47–1.60
– V3 (gain, broad)	−0.49	0.20	0.61	**	0.41–0.90	1.36	0.26	3.89	***	2.33–6.48
– V4 (loss, narrow)	0.37	0.21	1.45	*	0.97–2.16	−0.27	0.32	0.76		0.41–1.41
– V5 (loss, broad)	0.10	0.20	1.11		0.74–1.64	1.66	0.26	5.26	***	3.17–8.69
Sex (ref.: female)	0.61	0.14	1.85	***	1.39–2.44	0.81	0.18	2.25	***	1.57–3.22
Age (continuous variable)	−0.05	0.01	0.96	***	0.93–0.97	−0.05	0.01	0.96	***	0.92–0.98
Education (ref.: primary)				***					***	
– secondary	0.68	0.16	1.97	***	1.43–2.69	0.63	0.23	1.90		1.21–2.97
– higher	1.08	0.17	2.93	***	2.08–4.12	1.08	0.23	2.95	***	1.87–4.64
Marital status (ref.: without a partner)	−0.38	0.15	0.69	**	0.50–0.92	Variable not included in the model				
Constant	2.01	0.59	7.48	***		−0.82	0.74	0.44		
Significance of the model (Omnibus tests of model coefficients *p*-value)	0.000					0.000				
−2 Log likelihood	1381.54					956.43				
Cox and Snell R2	0.080					0.131				
Nagelkerke R2	0.107					0.204				
Hosmer and Lemeshow test (*p*-value)	0.311					0.157				

SE—standard error; CI—confidence interval; OR—odds ratio; Sig.—significance (in rows with the reference values it tells if the overall variable is significant in the model); * *p* < 0.1; ** *p* < 0.05; ***p < 0.01.

**Figure A1:**
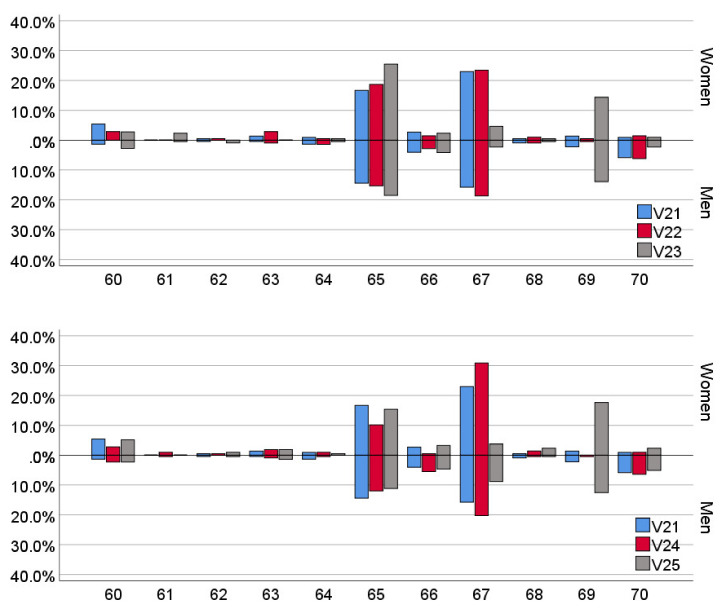
Impact of framing effect on planned retirement age by sex of respondents.

**Figure A2:**
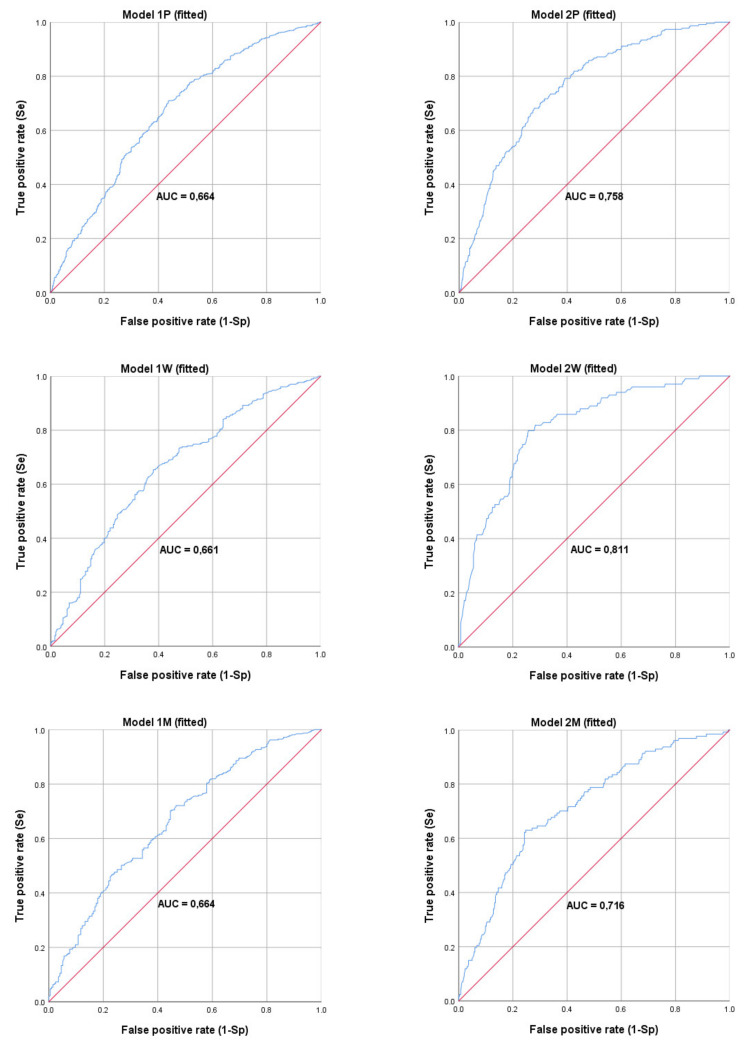
Receiver operating characteristic (ROC) curves for estimated models. Note: blue line—ROC curve; red line—reference line (line of classifications made by the random model); AUC—area under curve.

## Data Availability

The data presented in this study are available on request from the corresponding author.

## Appendix A

**Table A1:** The Wording of Different Variants of Vignette Questions.

Question Variant	Exact Wording of the Question
Male Respondents	
V1	Jan is considering when it is best to retire in the proposed pension system. He learned that if he retires at **65** years he will receive a benefit of 2000 PLN net per month (paid for life). If he decides to retire at the age of **63** years he will receive a benefit lower by approx. 350 PLN (1650 PLN). If he decides to retire at the age of **67** years, he will receive a benefit higher by approx. 420 PLN (2420 PLN). At what age would you have retired in Jan’s place?
V2	Jan is considering when it is best to retire in the proposed pension system. He learned that if he retires at **63** years he will receive a benefit of 1650 PLN net per month (paid for life). If he decides to retire at the age of **65** years he will receive a benefit higher by approx. 350 PLN (2000 PLN). If he decides to retire at the age of **67** years, he will receive a benefit higher by approx. 770 PLN (2420 PLN). At what age would you have retired in Jan’s place?
V3	Jan is considering when it is best to retire in the proposed pension system. He learned that if he retires at **61** years he will receive a benefit of 1370 PLN net per month (paid for life). If he decides to retire at the age of **65** years he will receive a benefit higher by approx. 630 PLN (2000 PLN). If he decides to retire at the age of **69** years, he will receive a benefit higher by approx. 1560 PLN (2930 PLN). At what age would you have retired in Jan’s place?
V4	Jan is considering when it is best to retire in the proposed pension system. He learned that if he retires at **67** years he will receive a benefit of 2420 PLN net per month (paid for life). If he decides to retire at the age of **65** years he will receive a benefit lower by approx. 420 PLN (2000 PLN). If he decides to retire at the age of **63** years, he will receive a benefit lower by approx. 770 PLN (1650 PLN). At what age would you have retired in Jan’s place?
V5	Jan is considering when it is best to retire in the proposed pension system. He learned that if he retires at **69** years he will receive a benefit of 2930 PLN net per month (paid for life). If he decides to retire at the age of **65** years he will receive a benefit lower by approx. 930 PLN (2000 PLN). If he decides to retire at the age of **61** years, he will receive a benefit lower by approx. 1560 PLN (1370 PLN). At what age would you have retired in Jan’s place?
Female Respondents	
V1	Barbara is considering when it is best to retire in the proposed pension system. She learned that if she retires at **65** years she will receive a benefit of 2000 PLN net per month (paid for life). If she decides to retire at the age of **63** years she will receive a benefit lower by approx. 350 PLN (1650 PLN). If she decides to retire at the age of **67** years, she will receive a benefit higher by approx. 420 PLN (2420 PLN). At what age would you have retired in Barbara’s place?
V2	Barbara is considering when it is best to retire in the proposed pension system. She learned that if she retires at **63** years she will receive a benefit of 1650 PLN net per month (paid for life). If she decides to retire at the age of **65** years she will receive a benefit higher by approx. 350 PLN (2000 PLN). If she decides to retire at the age of **67** years, she will receive a benefit higher by approx. 770 PLN (2420 PLN). At what age would you have retired in Barbara’s place?
V3	Barbara is considering when it is best to retire in the proposed pension system. She learned that if she retires at **61** years she will receive a benefit of 1370 PLN net per month (paid for life). If she decides to retire at the age of **65** years she will receive a benefit higher by approx. 630 PLN (2000 PLN). If she decides to retire at the age of **69** years, she will receive a benefit higher by approx. 1560 PLN (2930 PLN). At what age would you have retired in Barbara’s place?
V4	Barbara is considering when it is best to retire in the proposed pension system. She learned that if she retires at **67** years she will receive a benefit of 2420 PLN net per month (paid for life). If she decides to retire at the age of **65** years she will receive a benefit lower by approx. 420 PLN (2000 PLN). If she decides to retire at the age of **63** years, she will receive a benefit lower by approx. 770 PLN (1650 PLN). At what age would you have retired in Barbara’s place?
V5	Barbara is considering when it is best to retire in the proposed pension system. She learned that if she retires at **69** years she will receive a benefit of 2930 PLN net per month (paid for life). If she decides to retire at the age of **65** years she will receive a benefit lower by approx. 930 PLN (2000 PLN). If she decides to retire at the age of **61** years, she will receive a benefit lower by approx. 1560 PLN (1370 PLN). At what age would you have retired in Barbara’s place?

## Appendix B

**Table A2:** Average Planned Retirement Age Under Different Framing Scenarios.

Scenario	V1	V2	V3	V4	V5
Total					
N	222	209	216	217	215
Mean	65,94	66,11	66,10	66,13	66,51
Standard deviation	2.24	1.92	2.55	2.14	2.72
Men					
N	104	98	100	107	101
Mean	66.41	66.55	66.26	66.34	66.78
Standard deviation	2.10	1.75	2.60	2.22	2.50
Women					
N	118	111	116	110	114
Mean	65.53	65.72	65.96	65.94	66.27
Standard deviation	2.28	1.99	2.51	2.05	2.89

Note: To avoid gender bias the participating men were shown variants of the question with a male name and corresponding pronouns, and women with a female name and pronouns (see Table A1 in Appendix A).

**Table A3:** Logistic Regression Models of Planned Retirement Age (Women).

Variable	Model 1W					Model 2W				
	(>65 Years)					(>67 Years)				
	β	SE β	OR	Sig.	95% CI for OR	β	SE β	OR	Sig.	95% CI for OR
Framing effect (ref.: V1 (neutral))				**					***	
– V2 (gain, narrow)	−0.07	0.27	0.93		0.54–1.58	0.04	0.60	1.04		0.32–3.35
– V3 (gain, broad)	−0.44	0.27	0.65		0.37–1.09	2.12	0.47	8.35	***	3.31–21.0
– V4 (loss, narrow)	0.57	0.28	1.76	**	1.01–3.06	−0.12	0.62	0.89		0.26–3.01
– V5 (loss, broad)	0.05	0.27	1.05		0.61–1.79	2.66	0.47	14.27	***	5.73–35.5
Age (continuous variable)	−0.05	0.02	0.95	***	0.92–0.97	−0.05	0.02	0.95	**	0.90–0.98
Education (ref.: primary)				***					*	
– secondary	0.59	0.22	1.80	***	1.16–2.79	0.19	0.34	1.21		0.61–2.36
– higher	0.92	0.24	2.51	***	1.56–4.01	0.71	0.35	2.03	**	1.02–3.99
Marital status (ref.: without a partner)	−0.36	0.20	0.70	*	0.46–1.03	Variable not included in the model				
Constant	2.35	0.81	10.48	***		−0.66	1.16	0.52		
Significance of the model	0.000					0.000				
−2 Log likelihood	739.78					416.13				
Cox and Snell R2	0.077					0.176				
Nagelkerke R2	0.103					0.291				
Hosmer and Lemeshow test (*p*-value)	0.356					0.833				

SE—standard error; CI—confidence interval; OR—odds ratio; Sig.—significance (in rows with the reference values it tells if the overall variable is significant in the model); * *p* < 0.1; ** *p* < 0.05; *** *p* < 0.01.

**Table A4:** Logistic Regression Models of Planned Retirement Age (Men).

Variable	Model 1M					Model 2M				
	(>65 Years)					(>67 Years)				
	β	SE β	OR	Sig.	95% CI for OR	β	SE β	OR	Sig.	95% CI for OR
Framing effect (ref.: V1 (neutral))				*					***	
– V2 (gain, narrow)	0.02	0.30	1.02		0.56–1.85	−0.27	0.38	0.76		0.36–1.60
– V3 (gain, broad)	−0.54	0.30	0.58	*	0.32–1.03	0.88	0.34	2.41	***	1.24–4.65
– V4 (loss, narrow)	0.16	0.30	1.17		0.65–2.11	−0.41	0.38	0.67		0.31–1.39
– V5 (loss, broad)	0.19	0.31	1.21		0.66–2.19	0.90	0.33	2.47	***	1.28–4.73
Age (continuous variable)	−0.04	0.02	0.96	**	0.93–0.99	−0.04	0.02	0.96	**	0.92–0.99
Education (ref.: primary)				***					***	
– secondary	0.79	0.23	2.21	***	1.39–3.48	0.93	0.31	2.54	***	1.38–4.65
– higher	1.23	0.26	3.42	***	2.04–5.72	1.36	0.32	3.88	***	2.07–7.25
Health condition (ref.: bad)	Variable not included in the model								*	
– average						0.52	0.35	1.68		0.84–3.31
– good						0.03	0.36	1.03		0.51–2.08
Number of children (ref. 0)				*		Variable not included in the model				
– 1	−0.44	0.28	0.64		0.36–1.12					
– 2	−0.56	0.26	0.57	**	0.33–0.95					
– 3 or more	−0.76	0.34	0.47	**	0.23–0.91					
Constant	2.21	0.96	9.10	**		−0.26	1.11	0.77		
Significance of the model	0.000					0.000				
−2 Log likelihood	635.30					516.47				
Cox and Snell R2	0.081					0.104				
Nagelkerke R2	0.110					0.154				
Hosmer and Lemeshow test (*p*-value)	0.229					0.940				

SE—standard error; CI—confidence interval; OR—odds ratio; Sig.—significance (in rows with the reference values it tells if the overall variable is significant in the model); * *p* < 0.1; ** *p* < 0.05; *** *p* < 0.01.

## Appendix C

**Table A5:** Accuracy, Sensitivity and Specificity of the Models.

Model	Accuracy (Ac) [%]	Specificity (Sp) [%]	Sensitivity (Se) [%]
1P	64.4	42.6	80.5
2P	78.8	96.0	13.7
1W	62.7	53.2	70.9
2W	83.8	97.9	17.2
1M	65.7	33.3	85.7
2M	75.3	94.0	18.9

Note: the split point was set at 0.5; $Ac=\frac{TN+TP}{TN+TP+FN+FP}$; $Se=\frac{TP}{TP+FN}$; $Sp=\frac{TN}{TN+FP}$; where: TP—true positives; TN—true negatives; FP—false positives; FN—false negatives.
